# Wilkie's Syndrome: A Rare Clinical Case of Frailty Cachexia

**DOI:** 10.7759/cureus.90272

**Published:** 2025-08-17

**Authors:** Marta Roldão, Andreia Salgadinho Machado, João Oliveira, Marta Anastácio, Catarina Rodrigues

**Affiliations:** 1 Internal Medicine, Hospital de São Francisco Xavier, Lisbon, PRT; 2 Internal Medicine, Centro Hospitalar de Lisboa Ocidental, Lisbon, PRT

**Keywords:** duodenal obstruction, frailty syndrome, superior mesenteric artery syndrome, weight loss, wilkie syndrome

## Abstract

Superior mesenteric artery syndrome (SMAS) or Wilkie’s syndrome is a rare cause of gastrointestinal obstruction due to a compression of the duodenum between the superior mesenteric artery and the aorta, typically following significant weight loss, occurring more frequently in young women. We report a case of a 92-year-old female patient who presented with abdominal pain and vomiting associated with an unexplained weight loss in the last weeks. SMAS was diagnosed by computerized tomography, and conservative measures were taken, with the patient eventually dying. The outcome of this clinical case emphasizes the necessity of an early diagnosis and the need for an increasing awareness, mainly in patients with significant weight loss in order to prevent adverse outcomes.

## Introduction

Superior mesenteric artery syndrome (SMAS) or Wilkie’s syndrome is caused by upper gastrointestinal obstruction due to an acute angulation of the superior mesenteric artery (SMA) that compresses the third part of the duodenum between the SMA and the aorta [1]. It is a rare condition with only 500 cases described in the literature, with an estimated incidence between 0.013% and 0.3% [2,3].

The etiologic factor of SMAS is a significant weight loss that leads to a decrease in perimesenteric and retroperitoneal fat. There are also congenital and acquired anatomical variations that can contribute to the occurrence of this condition [1]. The clinical signs include nausea, vomiting, abdominal pain, anorexia, and weight loss. The severity of clinical symptoms is generally proportional to the degree of duodenal compression, with more pronounced compression often leading to more severe gastrointestinal manifestations.

The diagnosis criteria include an aortomesenteric angle (AMA) of less than 22° and an aortomesenteric distance of less than 8-10 mm [4]. A computerized tomography (CT) angiography is currently favored in the literature for diagnosis as it can reveal the narrowed AMA and distance and the extent of duodenal obstruction [1].

The SMAS is a potentially life-threatening condition, since the delay in diagnosis and treatment can lead to severe electrolyte imbalance, catabolic wasting, peritonitis, and visceral perforation. The treatment usually begins with conservative measures, consisting of enteral or parenteral nutrition in order to increase weight and restoring mesenteric fat to increase the AMA [5]. When this approach is not successful, surgery becomes mandatory in order to resolve the clinical condition [1]. In this publication, we report a severe case of SMAS in an elderly woman.

## Case presentation

**Figure 1:**
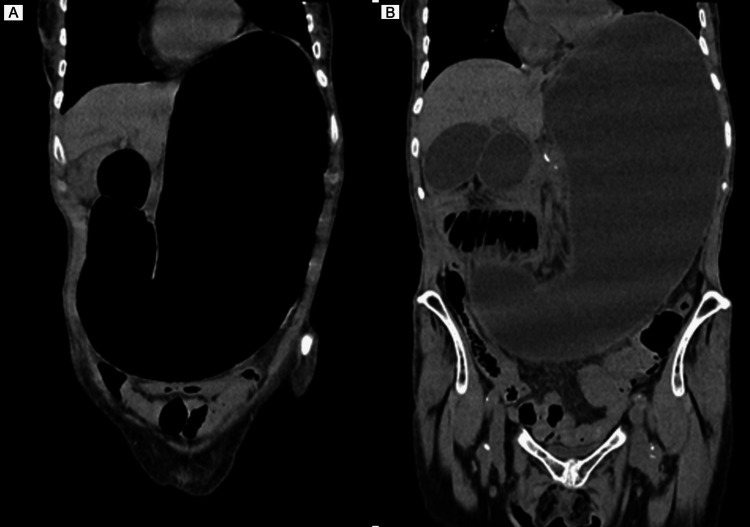
(A, B) Coronal view of abdominal CT showing gastroduodenal distention of the stomach until the third part of the duodenum. CT: computed tomography

**Figure 2:**
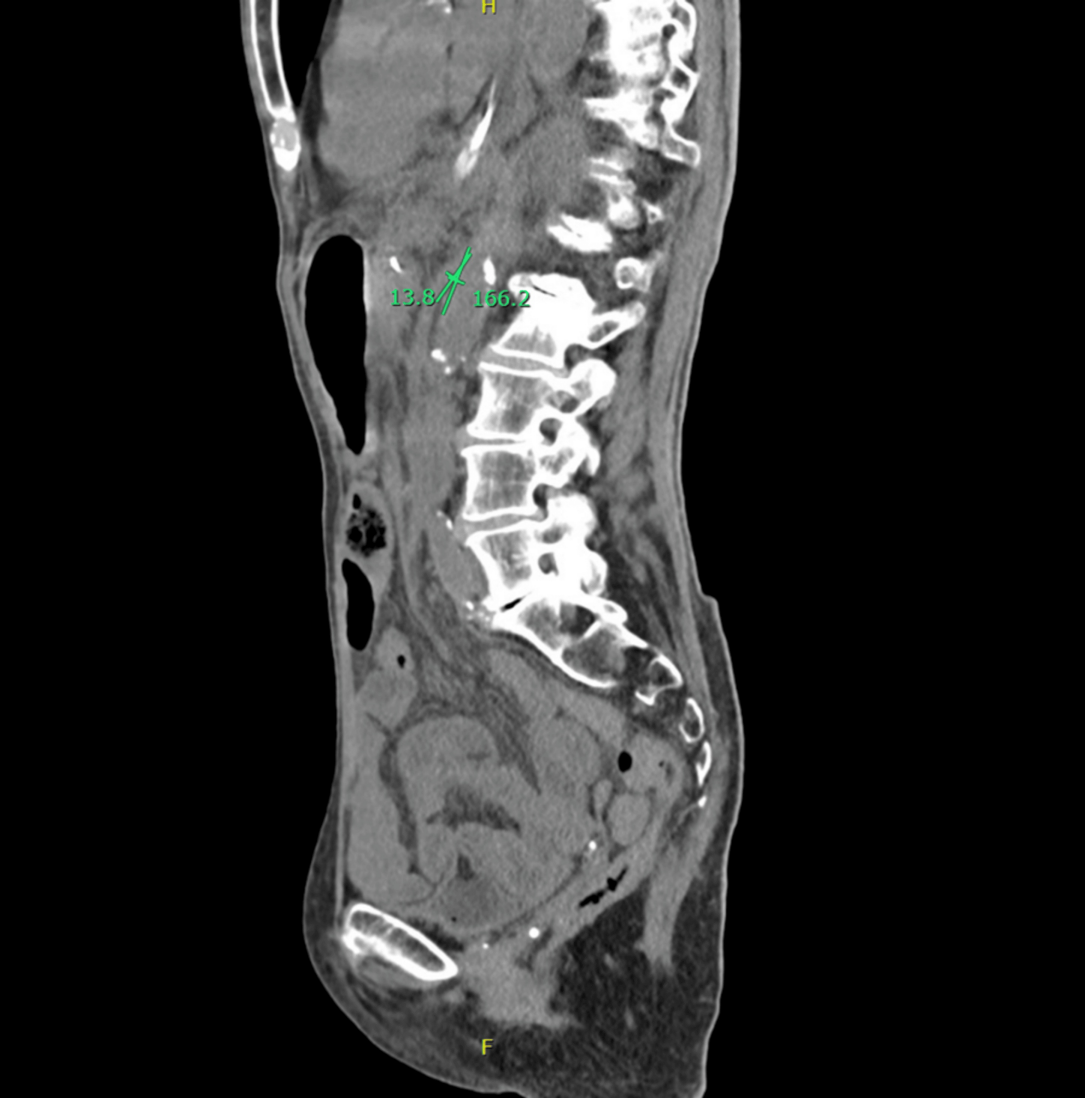
Sagittal view of abdominal CT showing a decrease in the aortomesenteric angle of 14°. CT: computed tomography

A frail 92-year-old female patient with a past medical history of Meniere’s disease, previous myocardial infarction, hypertension, and chronic atrial fibrillation presented to the emergency department with a one-week history of persistent nausea, non-bilious vomiting, and diffuse abdominal pain. She also reported an unintentional and significant weight loss over the past month. There was no history of recent changes in medication, gastrointestinal bleeding, altered bowel habits, or prior abdominal surgery. At admission, the vital signs were as follows: blood pressure 116/82 mmHg, pulse 121 bpm, afebrile, and a normal respiratory rate. On physical examination, the patient was awake and oriented, emaciated, and with severe cachexia. The abdominal examination revealed normal bowel sounds and diffuse abdominal pain. Laboratory testing showed normocytic normochromic anemia (hemoglobin (Hb) 12.1g/dL), white cell count 13,200/mm, C-reactive protein negative, stage II acute renal failure according to the Kidney Disease Improving Global Outcomes (KDIGO) with creatinine 1.40 mg/dL, urea nitrogen 61 mg/dL, elevated lipase and amylase (lipase 219 U/L, amylase 223 U/L), and HIV serology negative. An abdominal CT showed gastroduodenal distension of the stomach until the third part of the duodenum without intestinal pneumatosis or suggestive signs of pancreatitis (Figure 1). Conservative measures were initiated, and a nasogastric tube was placed with the removal of 2 L of gastric stasis. Repeat CT revealed a decrease in the aortomesenteric distance with an anteroposterior diameter of up to 3 mm, leading to a significant decrease in the third duodenal portion caliber. In the sagittal section, a decrease in the AMA of 14° was measured, which made the diagnosis of SMAS (Figure 2). Faced with the failure of refractory response to medical treatment, duodenojejunostomy was proposed, but she refused. Low-volume diet, fluid therapy, prokinetic agents, and parenteral feeding were initiated. Despite these measures, the patient maintained severe hypokalemia and hypernatremia and developed aspiration pneumonia. Due to the lack of clinical improvement with initial conservative measures, a nasojejunal tube was placed. Unfortunately, the patient passed away a few days later. The aspiration pneumonia was considered the cause of death.

## Discussion

This case presented an older female patient who does not represent a typical patient with SMAS. SMAS is more prevalent among women under 40 years old. Acquired causes cover a wide variety of etiologies that may predispose to the development of this syndrome: neoplasms, malabsorption syndromes, anorexia nervosa, HIV, serious injuries that imply prolonged immobilization, and post-operative status. The patient denied recent surgery or immobilization, and there were no signs of an occult neoplasm; thus, progressive sarcopenia and cachexia related to advanced age and frailty could not be excluded.

The symptoms of SMAS are non-specific, and they can occur for years or decades. The presentation can range from bloating and nausea to intestinal obstruction. The acute form occurs in a minority of patients and is associated with higher complications such as aspiration and severe pneumonia. Other complications resulting from persistent vomiting are dehydration with severe hypovolemia, oliguria, hydroelectrolytic alterations such as hypokalemia and metabolic alkalosis, and gastrointestinal bleeding.

There is no consensus on the preferred treatment for SMAS. The agreement among authors is that the treatment options should be based on the severity of the disease, using conservative measures as the first line of therapy [5]. Duodenojejunostomy is the preferred surgical approach when conservative management fails or in severe cases. This was a severe case refractory to conservative management. The surgical treatment was proposed but not accepted by the patient.

## Conclusions

Although SMAS is a rare condition, it can be life-threatening and requires prompt diagnosis and appropriate management. Clinicians should consider SMAS in the differential diagnosis of patients presenting with signs of duodenal obstruction-particularly those with recent or unexplained weight loss, regardless of age. While SMAS traditionally affects younger individuals, the growing prevalence of sarcopenia, frailty, and malnutrition among the elderly makes this population increasingly susceptible.

This case highlights the importance of maintaining a high index of suspicion, especially in older patients with non-specific gastrointestinal symptoms and significant weight loss. Early recognition and timely intervention are essential to avoid complications such as aspiration pneumonia, electrolyte imbalance, or gastrointestinal perforation. When conservative measures fail, surgical options like duodenojejunostomy should be promptly considered. Increased clinical awareness and familiarity with this rare syndrome can significantly improve outcomes through earlier diagnosis and targeted management.
